# Seismic Performance of Composite Shear Walls Constructed Using Recycled Aggregate Concrete and Different Expandable Polystyrene Configurations

**DOI:** 10.3390/ma9030148

**Published:** 2016-03-02

**Authors:** Wenchao Liu, Wanlin Cao, Jianwei Zhang, Qiyun Qiao, Heng Ma

**Affiliations:** College of Architecture and Civil Engineering, Beijing University of Technology, Beijing 100124, China; liuwenchao@emails.bjut.edu.cn (W.L.); zhangjw@bjut.edu.cn (J.Z.); qiaoqiyun@bjut.edu.cn (Q.Q.); maheng900611@sina.com (H.M.)

**Keywords:** recycled aggregate concrete, composite shear wall, seismic performance, expandable polystyrene modules, theoretical analysis, experimental study, numerical simulation

## Abstract

The seismic performance of recycled aggregate concrete (RAC) composite shear walls with different expandable polystyrene (EPS) configurations was investigated. Six concrete shear walls were designed and tested under cyclic loading to evaluate the effect of fine RAC in designing earthquake-resistant structures. Three of the six specimens were used to construct mid-rise walls with a shear-span ratio of 1.5, and the other three specimens were used to construct low-rise walls with a shear-span ratio of 0.8. The mid-rise and low-rise shear walls consisted of an ordinary recycled concrete shear wall, a composite wall with fine aggregate concrete (FAC) protective layer (EPS modules as the external insulation layer), and a composite wall with sandwiched EPS modules as the insulation layer. Several parameters obtained from the experimental results were compared and analyzed, including the load-bearing capacity, stiffness, ductility, energy dissipation, and failure characteristics of the specimens. The calculation formula of load-bearing capacity was obtained by considering the effect of FAC on composite shear walls as the protective layer. The damage process of the specimen was simulated using the ABAQUS Software, and the results agreed quite well with those obtained from the experiments. The results show that the seismic resistance behavior of the EPS module composite for shear walls performed better than ordinary recycled concrete for shear walls. Shear walls with sandwiched EPS modules had a better seismic performance than those with EPS modules lying outside. Although the FAC protective layer slightly improved the seismic performance of the structure, it undoubtedly slowed down the speed of crack formation and the stiffness degradation of the walls.

## 1. Introduction

Along with rapid urbanization, the construction industry has boomed significantly. As a result, construction waste has also increased tremendously. Removed concrete (construction waste) is often considered useless and disposed as demolition waste [1]. The reuse of discarded concrete has become a hot topic in the world leading to studies on waste concrete in many countries. Generally, the aggregate obtained from waste concrete by cleaning, crushing, and grading is called recycled concrete aggregate (RCA), and the concrete made from RCA is called recycled aggregate concrete (RAC). Since the 1950s, the possibility of RAC production from waste concrete has been studied [2]. In the 1970s, the United States reintroduced the use of RCA in nonstructural applications such as in filling materials, foundations, and base course materials [3]. In China, studies on RCA are still in their infancy. The country started to implement regulations [4] promulgated by the Ministry of Construction in 2005; the principle of construction waste reduction, harmless and resource-oriented environmental philosophy was proposed in the regulation. With the development of science and technology, more in-depth studies have been carried out. Kang *et al.* [5] conducted the flexural testing of reinforced concrete beams with RCAs; the results showed that the flexural behavior was not affected significantly by the use of up to 30% RCA. The fire performance of sustainable RCAs was studied by Gales *et al.* [6], and ten buildings were constructed using RCA concrete, thus playing an important role in the promotion of RCAs. RAC is no longer considered as a new technology in the world; so far, many studies have mainly focused on its applications [7,8,9,10,11,12].

Shear wall is one of the important structures that can bear a horizontal shear force; many studies have been carried out on shear walls [13,14,15,16,17]. With the increasing number of environmental problems in the world due to the construction industry, it has become necessary to use energy-efficient techniques for construction. Conventional shear walls cause several adverse effects on the environment; therefore, it is not appropriate to use them for constructions. Expanded polystyrene (EPS) composed of polystyrene, pentane, and a fire retardant has been used extensively because of its high chemical stability and excellent heat insulation property [18]. Therefore, new composite shear walls with energy conservation and heat preservation properties are highly desired. Wang *et al.* [19] conducted several tests on insulating masonry walls with eight different shear-span ratios and a solid wall to evaluate the seismic performance of the walls under a low-cycle repeated loading. Dou *et al.* [20] conducted four shear walls with different heights and width ratios of polystyrene insulation board, forming a heat insulation bearing integrated wall. Li studied the high shear-span ratio of glazed hollow bead insulation composite shear under a low cyclic loading [21]. Most of the previous studies were based on the combination of ordinary concrete and EPS modules, and mainly focused on the mechanical properties of the structure. However, studies on composite shear walls using a combination of recycled concrete and EPS module are rare. Cao *et al.* developed a new seismic-energy-saving shear wall structure using recycled concrete and EPS insulating cavity with a single row reinforcement net and surface mortar layer [22]. The combined single row of reinforcement and EPS heat preservation technology was applied to the recycled concrete shear wall. The surface mortar layer significantly affected the seismic performance of the shear wall. Because of the poor fire resistance of EPS modules, the fine aggregate concrete (FAC) outer protective layer was very necessary for the fire prevention in high-rise buildings. Therefore, it is very important to understand the behavior of a composite shear wall formed using EPS modules, recycled concrete, and FAC as the protective layer. In particular, the effect of a FAC protective layer and EPS module on the seismic performance of a composite wall should be evaluated.

Therefore, in this study, six recycled concrete wall specimens with EPS modules were designed for low-cycle reverse-loading tests. The effects of different shear-span ratios, EPS module layout, and FAC protective layer on the load-bearing capacity, stiffness, hysteresis characteristics, and seismic energy consumption of composite walls were analyzed, and the role of EPS thermal insulation modules and outer protection layer during the seismic process was verified. A calculation formula considering the role of FAC protective layer for the load-bearing capacity of the EPS module composite wall is proposed. Thus, this study provides a reference for the seismic design of this type of composite walls.

## 2. Experimental

### 2.1. Material Properties

**Figure 1:**
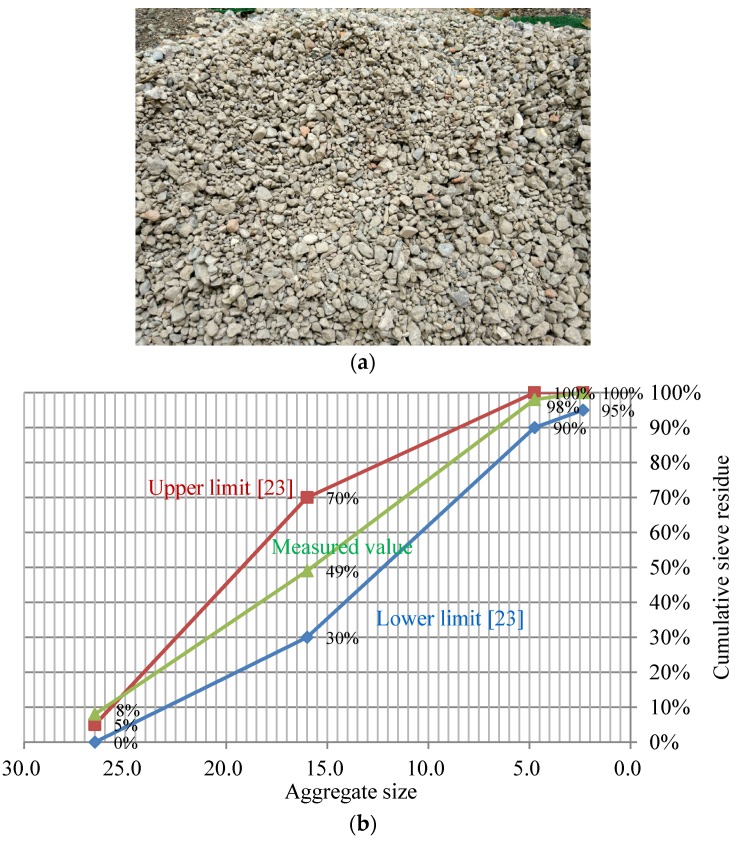
The aggregate gradation of the recycled aggregate concrete (RAC). (**a**) The coarse aggregate of RAC (aggregate size is 5–25 mm); (**b**) The sieve-curve of the coarse aggregate.

The RAC, used to prepare the specimens, was collected from an old building in Beijing, China. The discarded concrete was cleaned, crushed, and screened by grade before preparing the finely ground RCAs needed for the specimens as shown in Figure 1a. The RCA was tested according to GB/T 25177-2010 [23]; sieving, water absorption, and hazardous materials tests were carried out. Because the RCA was produced by demolition, components such as bricks were inevitable (~1.62 wt %). The RCA used in the study had an aggregate content of 40.1%, mortar aggregate content of 34.9%, residual mortar content of 23.42%, and toxic material content of 1.62%. The density of the RCA was 2290 kg/m^3^. Its water absorption capacity was 3.23%. Figure 1b shows the sieve-curve of the coarse aggregate in RCA.

According to JGJ 55-2011 [24], the material consumption amount needed by the recycled concrete was determined based on the mix proportion design method of ordinary concrete. The strength grade of the six recycled concrete specimens was RC40, with the designed mix proportion (cement:water:sand:recycled-pebble) = 1:0.83:4.01:4.01. To be specific, the replacement rate of the coarse aggregates in RAC was 100%, and the fine aggregate of RAC was ordinary sand. Moreover, according to JGJ 55-2011, FAC is a type of ordinary concrete, and the maximum size of coarse aggregate is less than 15 mm. The designed mix proportion (cement:water:sand:pebble) of the FAC was 1:0.58:2.46:3.27.

Table 1 shows the measured mean compressive strength (28 day) of cubes and mean elastic modulus of recycled concrete blocks [25]. Notably, all the tested blocks were naturally cured under the same conditions with the specimens produced in this study.

**Figure 2:**
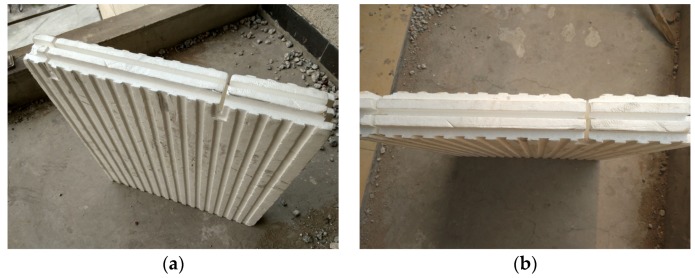
Expanded polystyrene (EPS) module. (**a**) Side elevation of EPS module; (**b**) Plan view of EPS module.

EPS is an excellent energy-saving and thermal insulation material that can be manufactured into EPS module components by industrialization processing [18]. EPS modules were assembled and lap-jointed together by tongue-and-groove modules as shown in Figure 2. The properties of the EPS module are listed in Table 2.

The rebar diameters used in walls were 4, 6, 8 and 12 mm. The measured mechanical properties of each type of steel are listed in Table 3, where *f_y_* is the yield strength, *f_u_* is the ultimate strength, δ is the ductility, and *E_s_* is the elastic modulus.

### 2.2. Details of Test Specimens

Six recycled concrete composite shear wall specimens with EPS modules were designed. Three of the six specimens were used to construct mid-rise walls with a shear-span ratio of 1.5, and other three were used to construct low-rise walls with a shear-span ratio of 0.8. The mid-rise and low-rise shear walls consisted of an ordinary recycled concrete shear wall, composite wall with an FAC protective layer (EPS modules external insulation layer), and composite wall with sandwiched EPS module insulation layer. The parameters of the specimens are listed in Table 4.

Specimens SW1.5-1 and SW0.8-1 were recycled concrete shear walls of 200 mm in thickness, and the web rebar of wall had double rows, double directions, and with a steel ratio of 0.25%. The reinforcement of the concealed columns at both the ends of the wall corner was 4D12, and the stirrup was D6-150 mm.

Specimens SW1.5-2 and SW0.8-2 were recycled concrete composite shear walls of 320 mm in thickness, including a FAC fire wall of 50 mm in thickness, EPS modules of 70 mm in thickness, a recycled concrete wall of 200 mm in thickness, and a single layer D4-10 mm bidirectional steel net arranged in the middle of the FAC. D6 connecting bars were used to connect the thermal insulation modules. The steel ratio in the recycled concrete wall was the same as that in specimens SW1.5-1 and SW0.8-1.

**Figure 3:**
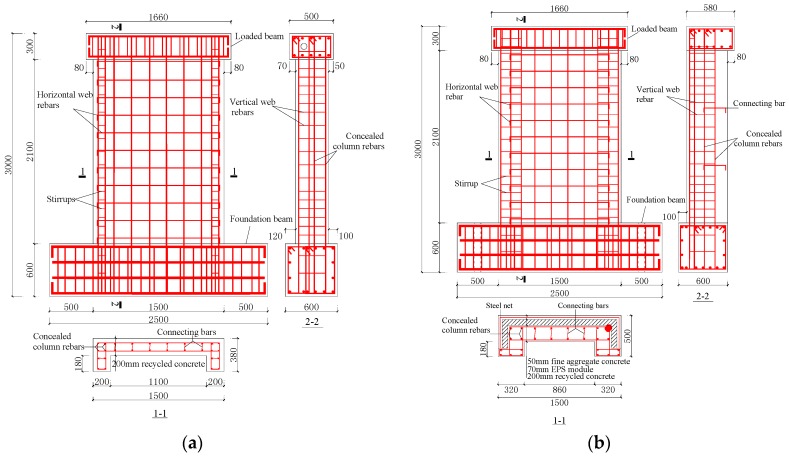

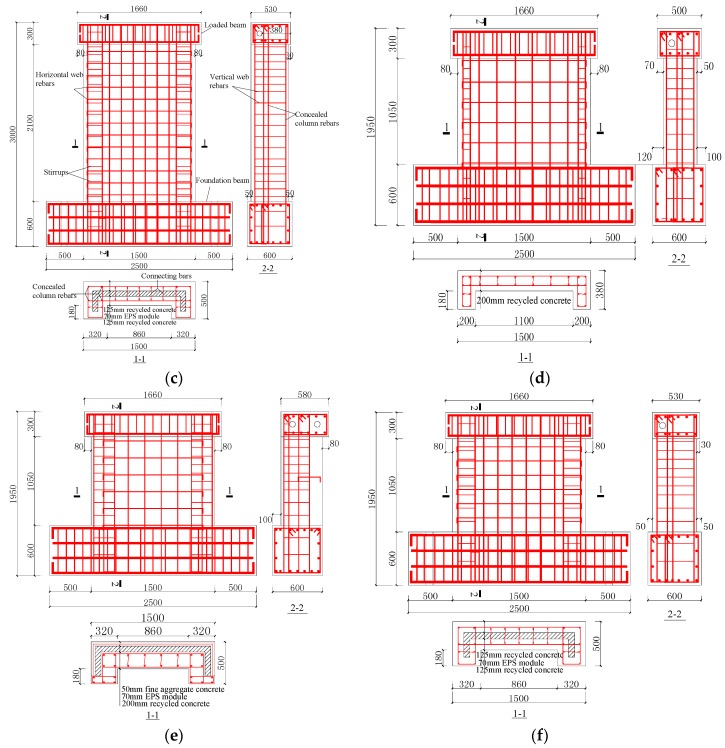
Detail information of the reinforcement of test specimens. (**a**) SW1.5-1; (**b**) SW1.5-2; (**c**) SW1.5-3; (**d**) SW0.8-1; (**e**) SW0.8-2; (**f**) SW0.8-3.

Specimens SW1.5-3 and SW0.8-3 were recycled concrete composite walls of 320 mm in thickness with EPS module and sandwiched thermal insulation, including EPS modules of 70 mm in thickness in the middle and a recycled concrete composite wall of 125 mm in thickness on both sides. The web rebar of the recycled concrete composite wall on both the sides of the modules was single-row bidirectional D8-160, see the detailed information about the reinforcement of the component in Figure 3.

### 2.3. Construction of Test Specimens

**Figure 4:**
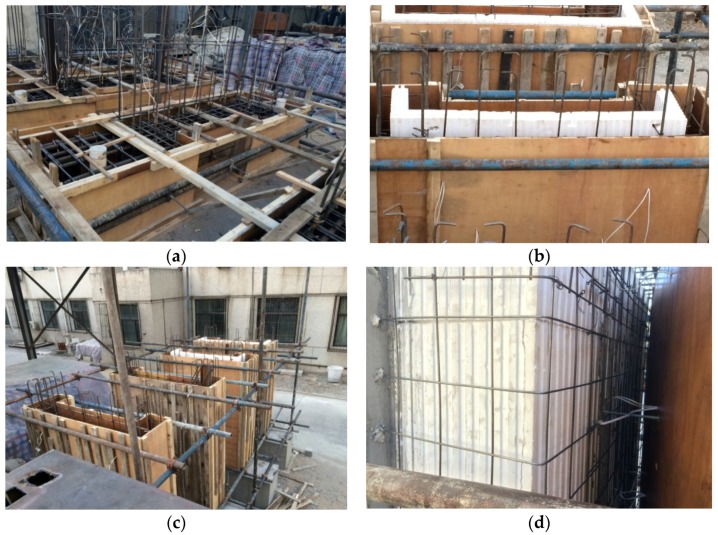
Preparation of specimens. (**a**) Lashing foundation reinforcement; (**b**) Assembling EPS module; (**c**) Pouring recycled concrete; (**d**) Pouring fine aggregate concrete (FAC).

The sample was prepared outside the laboratory of Beijing University of Technology (Beijing, China); the detailed preparation steps were as follows: (1) Binding the foundation reinforcing bars, followed by positioning and binding column bars and vertical reinforcement in the wall; (2) pouring foundation concrete; (3) joining the EPS thermal insulation modules, followed by strapping horizontal reinforcement and steel hoops; (4) pouring the recycled concrete wall and FAC as the outer fire protection layer, and making the test module; (5) strapping loading beam steel, and pouring loading beam concrete. Figure 4 shows the preparation of the specimens.

### 2.4. Test Set-Up and Loading Programme

**Figure 5:**
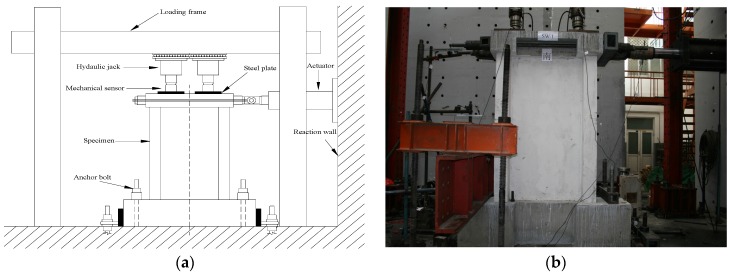
Test set-up. (**a**) Loading device; (**b**) Loading field.

The repeated low-cycle loading method was used in the experiments. First, a 1600 kN vertical load with an axial compression ratio of 0.15 was applied on the loading beam of the specimens, controlled by two vertical jacks with a measuring range of 1000 kN. Then, the horizontal load was applied through the horizontal tension and compression of the jacks; the distances (*h*) from the horizontal loading points to the foundation top surface of the specimen were 2250 mm (mid-rise wall with a shear-span ratio of 1.5) and 1200 mm (low-rise wall with a shear-span ration of 0.8), respectively. The loading device is shown in Figure 5.

**Figure 6:**
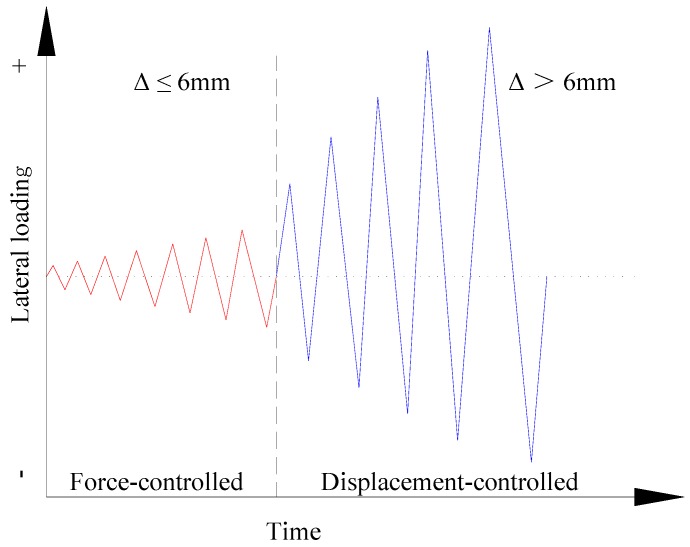
Loading history of specimens.

The horizontal load was applied using a combined control of load and displacement. For the wall with a shear-span ratio of 1.5, in the first stage, the load was controlled by the force, and when the horizontal displacement attained ($\Delta =\frac{1}{375}h=\frac{1}{375}\times 2250\leq 6\;mm$), the force was applied with an increase of 30 kN force per cycle. In the second stage, a displacement controlled loading protocol with 1/750 displacement angle (θ) increment was used. On the other hand, for the wall with a shear-span ratio of 0.8, in the first stage, the load was controlled by the force, and, when the horizontal displacement attained ($\Delta =\frac{1}{200}h=\frac{1}{200}\times 1200\leq 6\;mm$), the force was applied with an increase of 30 kN force per cycle. In the second stage, a displacement controlled loading protocol with 1/600 displacement angle (θ) increment was used. The load course of the specimen is shown in Figure 6.

**Figure 7:**
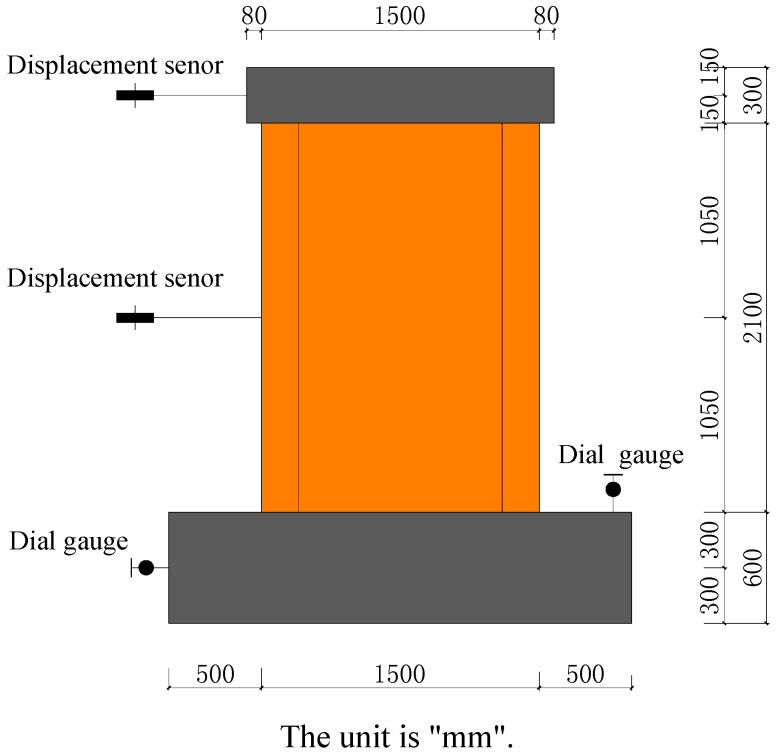
Test point arrangement of specimens.

Differential displacement sensors were installed at the horizontal loading point and at the central height of the wall. Dial gauges were installed on the side of the foundation and on the top surface of the foundation for monitoring the foundation horizontal slip and the vertical turn-up of the foundation, respectively. The displacement sensor, dial gauges, and IMP (Isolated Measurement Pods) data collection system were butt-jointed as shown in Figure 7. During the tests, the cracks and failure at each position were recorded through the visual observation of experimental phenomena.

## 3. Results and Discussion

### 3.1. Failure Characteristics

**Figure 8:**
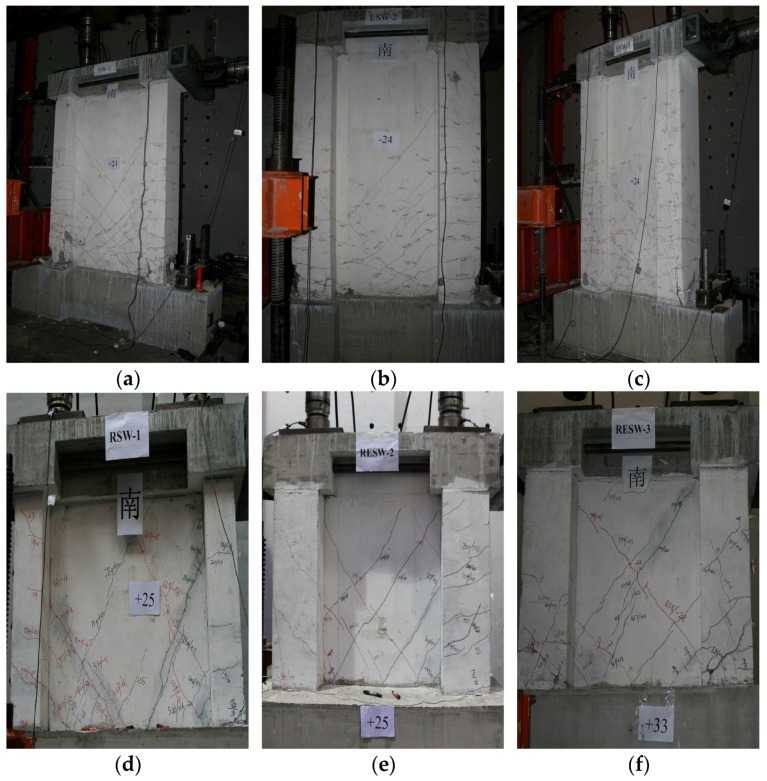
Failure characteristics of six walls. (**a**) SW1.5-1; (**b**) SW1.5-2; (**c**) SW1.5-3; (**d**) SW0.8-1; (**e**) SW0.8-2; (**f**) SW0.8-3.

When the displacement angle (θ) was ~1/50, the wall body with a shear-span ratio of 1.5 and that with a shear-span ratio of 0.8 failed. However, both the specimens showed different failure mechanisms, *i.e.*, the former specimen showed signs of bending failure, whereas the latter specimen failed in shear. This indicates that the shear-span ratio plays a significant role in affecting the failure mechanism of shear walls. Figure 8 shows the failure mechanism of each specimen.

The cracks on the specimens mainly appeared on both the flanges and on the web plates of the shear walls. Horizontal cracks appeared on the bottom as well as on the middle of the flanges, whereas 45° diagonal cracks and random diagonal cracks were observed on both sides of the web plates.

**Figure 9:**
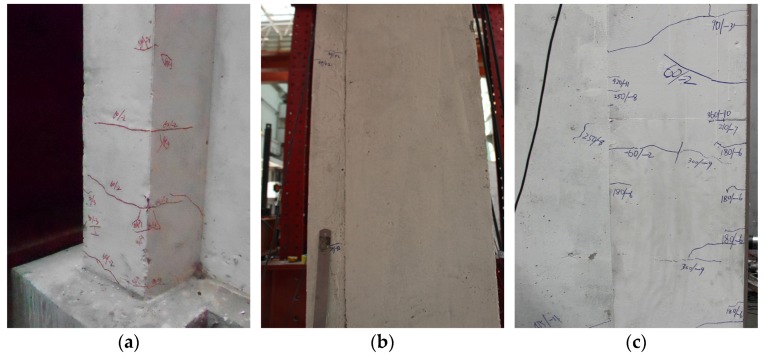
The first crack of specimens (shear-span ratio is 1.5). (**a**) SW1.5-1; (**b**) SW1.5-2; (**c**) SW1.5-3.

For the wall with a shear-span ratio of 1.5, the first crack appeared in the height range 200–300 mm of the south foundation top surface of the west flange for SW1.5-1, in the height range 600–700 mm of the east recycled concrete area of the east flange for SW1.5-2 and at the height of 830 mm of the south of the east flange for SW1.5-3 as shown in Figure 9. With the increase in horizontal load, the horizontal cracks on both the flanges of the shear walls increased.

**Figure 10:**
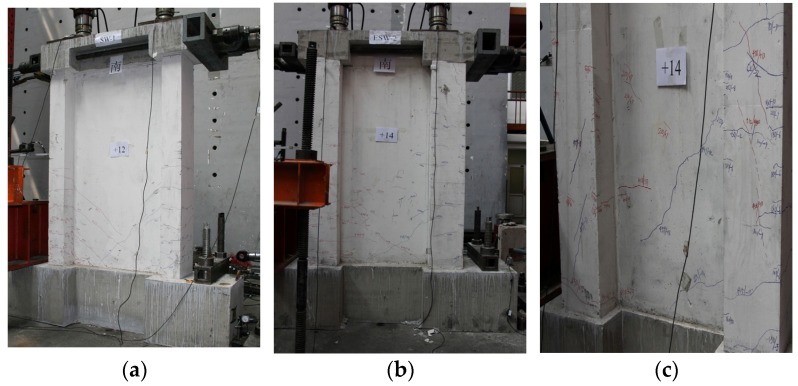
Web cracks at the middle stage of loading for specimens (shear-span ratio is 1.5). (**a**) SW1.5-1; (**b**) SW1.5-2; (**c**) SW1.5-3.

Figure 10a,c show that when the displacement angle (θ) of specimens SW1.5-1 and SW1.5-3 reached 1/1406 and 1/1573, respectively, 45° cracks started to appear at the web angle of the shear wall. When the loading was carried forward to the mid-term, many “X”-shaped cracks formed with the reciprocal chiasma of diagonal cracks at the web plates in SW1.5-1; however, the diagonal cracks at the web plates in SW1.5-3 developed relatively slowly. When the displacement angle (θ) of the SW1.5-2 specimen reached 1/300, first, slant 45° cracks appeared in the FAC layer north of the web plates of the wall. With the increase in the displacement angle, the diagonal cracks at the north of the web plates did not increase noticeably; when the displacement angle (θ) reached 1/809, slant 45° cracks started to appear in the FAC layer south of the web plates of the wall body. Slant cracks at the south of the web plates were clearly less than those in SW1.5-1, and only a few “X”-shaped cracks were observed as shown in Figure 10b. This indicates that the crack development can be slowed down by the protection layer outside the FAC.

**Figure 11:**
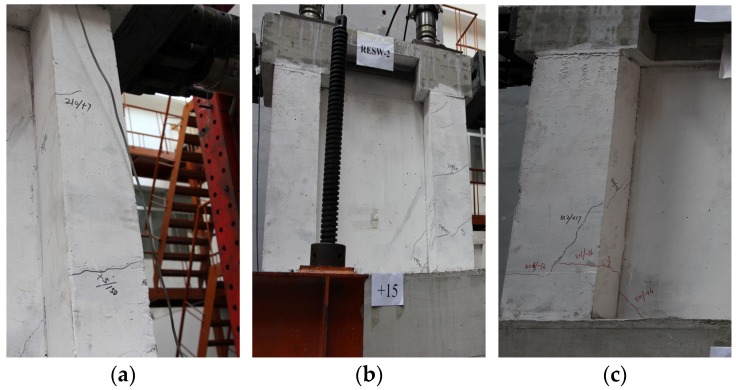
The first crack of specimens (shear-span ratio is 0.8). (**a**) SW0.8-1; (**b**) SW0.8-2; (**c**) SW0.8-3.

For the specimen with a shear-span ratio of 0.8, Figure 11a,b show that the first cracks in the SW0.8-1 and SW0.8-2 wall appeared at the north of east flange, 300–550 mm away from the foundation top surface. On the other hand, the cracks in the SW0.8-2 specimen appeared later compared to SW0.8-1 specimen. The first cracks in the SW0.8-3 specimen appeared at the south of the west flange, 250–300 mm away from the foundation, slightly earlier than that in SW0.8-1 as shown in Figure 11c. In the middle of the loading process, cut-through diagonal cracks appeared on the web plates of SW0.8-1, and diagonal inclined cracks appeared on the web plates of SW0.8-2 and SW0.8-3. Beyond this point, multiple “X”-shaped cracks appeared on the web plates of the SW0.8-1 wall, tightly close to both the flanges. For SW0.8-2, multiple diagonal cracks cut through the flanges on the web plates. Similarly, multiple “X”-shaped cracks appeared on the flanges and on the middle of the web plates in SW0.8-3.

**Figure 12:**
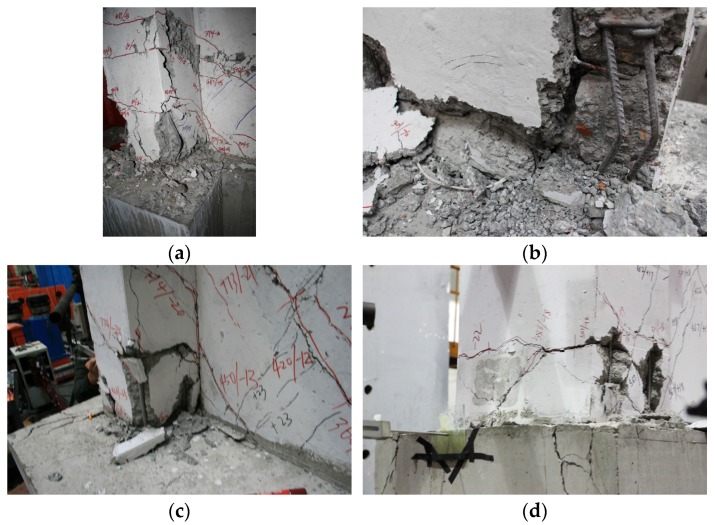
Failure details of specimens. (**a**) Reinforcement yielding (SW1.5-1); (**b**) Failure zone of concrete (SW1.5-3); (**c**) Concrete-flake-off (SW0.8-1); (**d**) Exposed reinforcement (SW0.8-3).

In terms of failure mode, the damages in the web plates and flanges of ordinary recycled concrete specimens (SW1.5-1 and SW0.8-1) were most severe, followed by the sandwich thermal insulation composite walls (SW1.5-3 and SW0.8-3) and composite walls with the EPS modules and outer protection layer (SW1.5-2 and SW0.8-2). The failure process of the specimens was as follows: The vertically distributed steel bars yielded first or even snipped, a part of the vertically steel bars yielded, a large amount of concrete at the bottom of the flanges crushed, and a continuous crushing failure band formed and stretched to the web plates of the wall body as shown in Figure 12.

### 3.2. Load-Bearing Capacity

**Figure 13:**
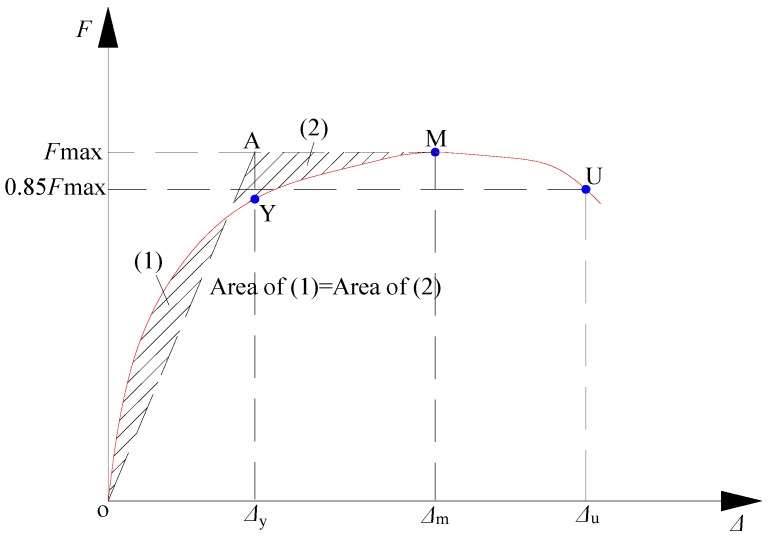
Determination of yield point.

Table 5 shows the cracking load *F_c_*, yield load *F_y_*, peak load *F_u_*, and their ratios (μ*_cu_* = *F_c_*/*F_u_*, μ*_yu_* = *F_y_*/*F_u_*). Each load has a measured value (*MV*) and relative value (*RV*). In this study, the cracking load was the load when the first visible cracks appeared, and the yield load was determined by the energy equivalence method [26] as shown in Figure 13. The loads were the mean absolute values of the positive and negative values.

Under the same configuration, the capacity of the specimens with a shear-span ratio of 0.8 was larger than that of those with a shear-span ratio of 1.5, indicating that when the shear-span ratio is smaller, the capacity of the component is higher. Compared to SW1.5-2, the yield strength of SW1.5-3 increased by 10.3%, and the peak load increased by 14.5%, indicating that the arrangement of the EPS modules in the middle of the shear wall is more beneficial for improving the capacity of recycled concrete composite shear walls than arranging them outside the wall. On the other hand, SW0.8-3 and SW0.8-2 had an almost equal peak load, indicating that the arrangement form of the EPS modules slightly affected the capacity of the shear walls. Compared to SW0.8-1, the yield strength of SW0.8-2 increased by 13.7%, and the peak load increased by 8.5%, indicating that the capacity of the walls was improved by the EPS modules and the outer protection layer of FAC.

### 3.3. Stiffness and Degradation

**Figure 14:**
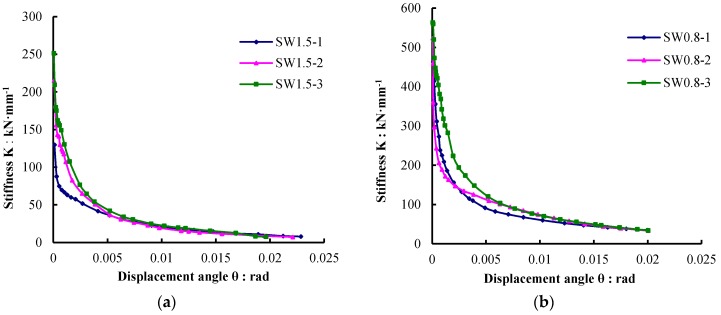
Stiffness degradation curves of specimens. (**a**) Shear-span ratio is 1.5; (**b**) Shear-span ratio is 0.8.

The secant stiffness of each specimen was computed from the skeleton curve of the hysteresis plot using Equation (1). *F* is the horizontal force, and the *U* is the horizontal displacement. The measured stiffness values and their degeneration factors are listed in Table 6. *K*_0_ is the initial elastic stiffness, and *K_y_* is the yield stiffness. The relationship curve “stiffness *K*-displacement angle θ” is shown in Figure 14.

(1) $$K_{i}=\frac{F_{i}}{U_{i}}$$

Under the same configuration, the stiffness of the specimens with a shear-span ratio of 0.8 was higher than those with a shear-span ratio of 1.5. Compared to SW1.5-1, the initial stiffness of specimen SW1.5-2 increased by 65.6%, and the yield stiffness increased by 113.8%. On the other hand, compared to SW0.8-1, the initial stiffness of SW0.8-2 increased by 13.1%, and the yield stiffness increased by 52.7%. This indicates that the EPS modules and protection layer outside the FAC improved the stiffness of composite shear walls and significantly reduced the rate of stiffness degeneration. By comparing the stiffness degeneration processes of SW1.5-2 and SW1.5-3 as well as SW0.8-2 and SW0.8-3, it was found that the arrangement of the EPS thermal insulation modules in the middle of the shear wall is more beneficial for improving the stiffness than arranging the EPS thermal insulation modules on one side of the wall.

### 3.4. Load-Displacement Response

**Figure 15:**
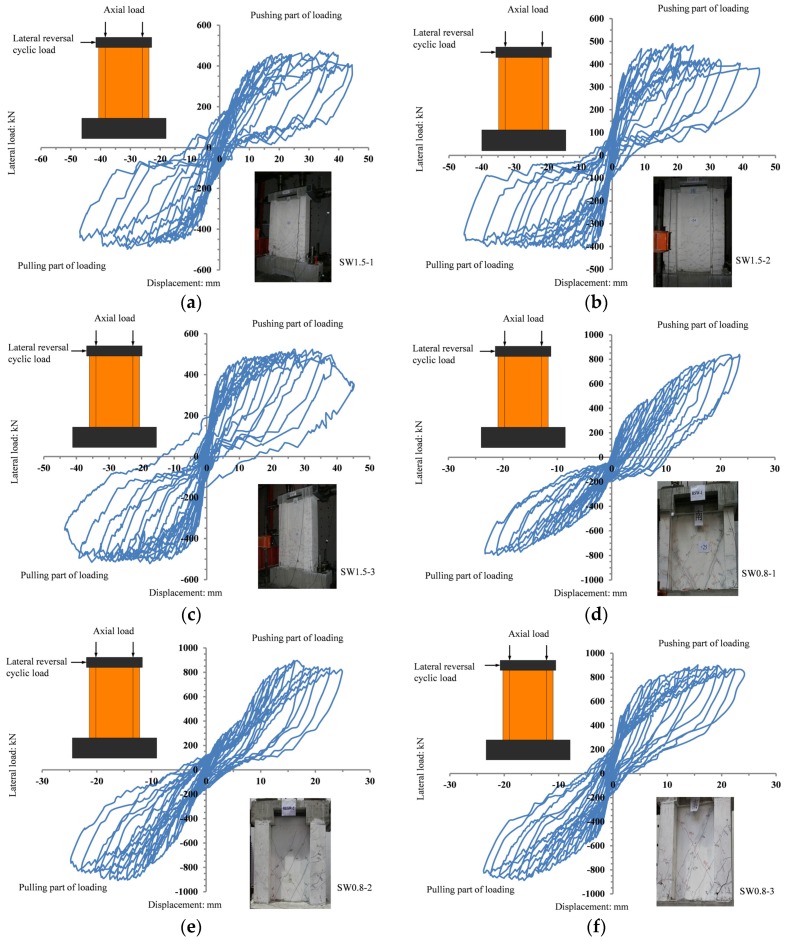
Hysteresis curves of specimens. (**a**) SW1.5-1; (**b**) SW1.5-2; (**c**) SW1.5-3; (**d**) SW0.8-1; (**e**) SW0.8-2; (**f**) SW0.8-3.

The horizontal load–displacement relationships of the specimens are shown in Figure 15. The ultimate displacement angle of ordinary shear walls was in the order of 1/120, and in this experiments, the maximum displacement angle was 1/50.

At the initial stage of the loading, the displacement increased linearly with the force, and the residual deformations after the unloading were negligible. With the increase in repeated cyclic loading, the peak displacement as well as the residual displacement increased gradually, whereas the stiffness degradation became evident until failure.

Compared to SW1.5-1, the residual strain of SW1.5-2 after the unloading became larger with a slightly smaller capacity. However, during the failure, the elastic-plastic deformation capacity and seismic-resistance and energy-dissipation capacity of the shear walls were improved by the combined effect of the EPS thermal insulation modules and FAC to some extent. Compared to SW1.5-2, the hysteresis loop of SW1.5-3 was full, and the capacity of the specimen improved. The adhesion between the EPS thermal insulation modules and the recycled concrete on both the sides of the EPS modules was better than that between the EPS thermal insulation modules and FAC. This not only improved the overall response of the specimen, but also enhanced the seismic-resistance and energy-dissipation capacity to a large extent.

On the other hand, compared to SW0.8-1, the hysteresis loop of SW0.8-2 was full, and the capacity improved. This indicates that the EPS modules and FAC participated satisfactorily in the seismic-resistance and energy-dissipation processes and improved the capacity of the specimens. Compared to SW0.8-2, the hysteresis loop of SW0.8-3 was fuller with a slow degradation of capacity, and its structural ductility was better. This indicates that the adhesion between sandwich-arranged EPS modules with recycled concrete on both sides of the modules was good, thus enhancing the overall response of the specimen and further improving the seismic response of the composite walls.

**Figure 16:**
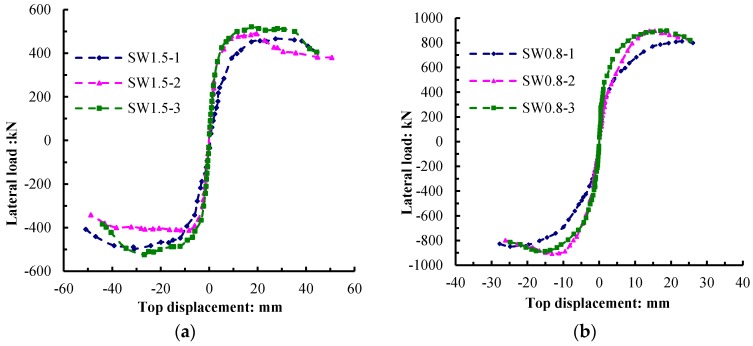
Force–displacement skeleton curves of the specimens. (**a**) Shear-span ratio is 1.5; (**b**) Shear-span ratio is 0.8.

Figure 16 shows a comparison of the force–displacement skeleton curves of the specimens with different shear-span ratios. Under different shear-span ratios, the capacity of the specimens with a shear-span ratio of 1.5 was lower than that of the specimens with a shear-span ratio of 0.8. For the specimens with the same shear-span ratio, the recycled concrete wall with the EPS modules arranged in the middle had the highest capacity, and the strength degradation was the slowest. Compared to SW1.5-1, the yield stiffness of SW1.5-2 was relatively large with a relatively low capacity. After the wall cracking, the stiffness degeneration rate of SW1.5-2 was higher than that of SW1.5-1. SW1.5-3 had a force–displacement skeleton curve similar to that of SW1.5-2, but both its initial stiffness and yield stiffness improved. With the increase in displacement angle, the strength degradation of SW1.5-3 was slower than that of SW1.5-2. Compared to SW0.8-1, the initial stiffness of SW0.8-2 was relatively small, with a relatively higher yield stiffness and larger capacity. Compared to SW0.8-2, the initial stiffness and yield stiffness of SW0.8-3 improved. With the increase in displacement angle, the descent stage of the load-bearing capacity of the specimens became smooth and steady with a good structural ductility.

### 3.5. Energy Dissipation Capacity

**Figure 17:**
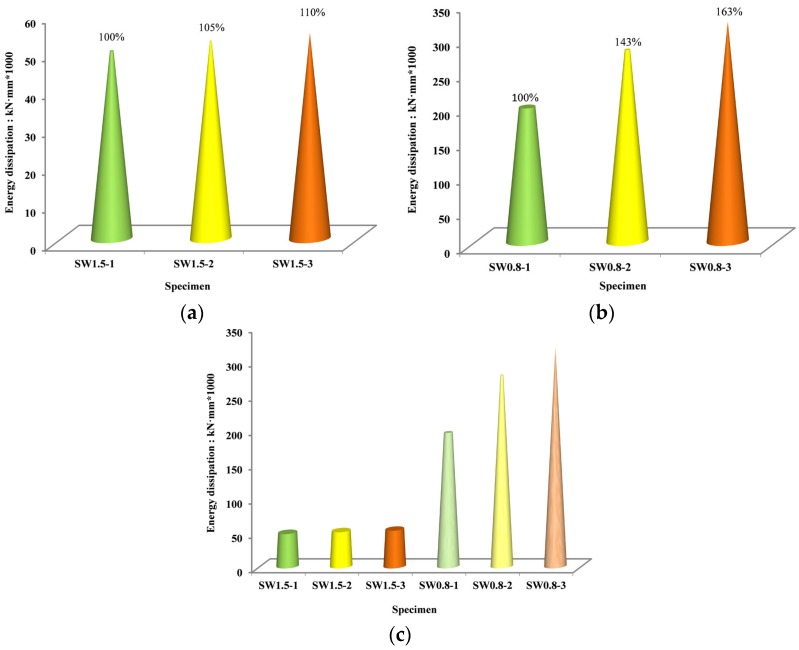
Energy dissipation histogram of specimens. (**a**) Energy dissipation for specimens (shear-span ratio is 1.5); (**b**) Energy dissipation for specimens (shear-span ratio is 0.8); (**c**) Energy dissipation of all specimens.

The area under the hysteresis curve of the specimen under the action of low-reversed cyclic loading shows the magnitude of seismic energy dissipated by the structure. In this study, the area surrounded by the skeleton curve (which was connected by the peak points of the hysteresis curve) of the specimen when the displacement angle reached 1/50 was used as the representative value of the energy dissipation of the shear wall. Table 7 lists the energy dissipation of each specimen, where E_0.02_ is the energy dissipation value when the displacement angle of the specimen was 1/50. The energy dissipation of each specimen is shown in Figure 17.

For the same configuration, the energy dissipation of the specimens with a shear-span ratio of 1.5 was lower than that of the specimens with a shear-span ratio of 0.8. This is because the capacity of the medium-high wall was relatively smaller. For the specimens with a shear-span ratio of 0.8, compared to SW0.8-1, the total energy dissipation of SW0.8-2 increased by 42.6%. This indicates that the EPS modules and their outer FAC participated satisfactorily in the seismic-resistance and energy-dissipation processes and accordingly enhanced the energy dissipation capacity of the walls significantly. However, when the shear-span ratio was 1.5, the contribution of the EPS modules and FAC on the total energy dissipation was not very significant. When comparing SW1.5-3 with SW1.5-2, and SW0.8-3 with SW0.8-2, their overall energy dissipations increased by 4.1% and 14.6%, respectively. This indicates that the arrangement form of the EPS modules significantly affected the seismic-resistance and energy-dissipation of recycled concrete shear walls, and that, when the shear-span ratio was small, the sandwich arrangement significantly enhanced the seismic performance.

## 4. Theoretical Analysis

### 4.1. Calculation of Load-Bearing Capacity

#### 4.1.1. Analysis of the Load-Bearing Capacity of the Normal Section

The experimental study showed that the shear wall with a shear-span ratio of 1.5 suffered bending failure, and the failure mode was controlled by the bending moment in the bottom of the shear wall. Thus, the load-bearing capacity of mid-rise walls could be calculated by the normal section. The load-bearing capacity calculation for the normal section should be consistent with the following basic assumptions:

(1) The section meets the assumption of the plane section.
(2) Neglect the tensile effect of recycled concrete and FAC in the tension zone.
(3) The maximum compressive stress of recycled concrete and FAC is taken from the standard values of axial compressive strength *f_ck,r_* and *f_ck,f_*.
(4) The double-fold line mode is used for the constitutive model of steel bar. The mode shows the linear elastic relationship before the steel bar yields, and the yield strength is used after the yielding.
(5) Ignore the vertical distribution force of reinforcement near the pressure area and neutral axis.
(6) Consider the tensile strength of the vertical bar in the range of $h_{w0}-1.5x$ [27], where *x* is the height of the concrete compression zone.

**Figure 18:**
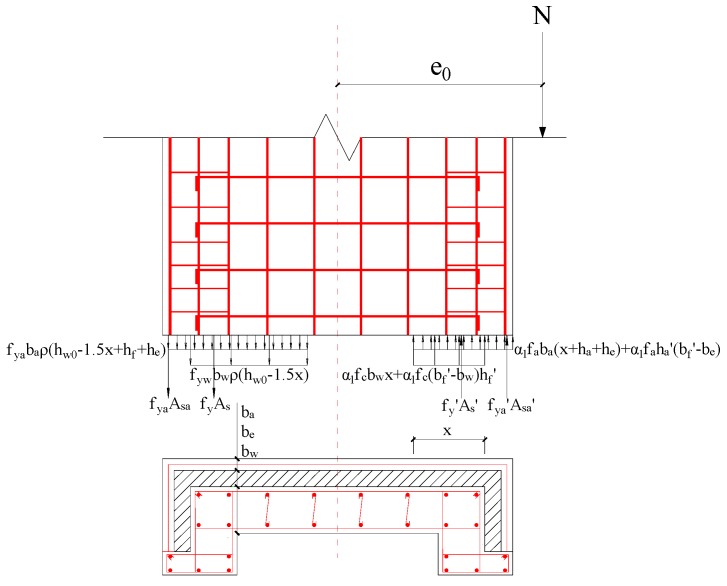
Mechanical model of load-carrying.

The calculation model for the load-bearing capacity of the normal section of the shear wall is shown in Figure 18. The calculation formula for the load-bearing capacity of the normal section is expressed by Equations (2) and (3).

(2) $$\begin{array}{cc} N & =\alpha_{1}f_{c}b_{w}x+\alpha_{1}f_{c}(b_{f}^{'}-b_{w})h_{f}^{'}+\alpha_{1}f_{a}b_{a}(x+h_{a}+h_{e})+\alpha_{1}f_{a}h_{a}^{'}(b_{f}^{'}+b_{e})+f_{y}^{'}A_{s}^{'}\\ & +f_{ya}^{'}A_{sa}^{'}-f_{y}A_{s}-f_{ya}A_{sa}-f_{yw}b_{w}\rho (h_{w0}-1.5x)-f_{ya}b_{a}\rho (h_{w0}-1.5x+h_{f}+h_{e}) \end{array}$$

(3) $$\begin{array}{cc} Ne & =\alpha_{1}f_{c}b_{w}x(h_{w0}-\frac{x}{2})+\alpha_{1}f_{c}h_{f}^{'}(b_{f}^{'}-b_{w})(h_{w0}-a_{s})+\alpha_{1}f_{a}b_{a}(x+h_{a}+h_{e})(h_{w0}-\frac{x}{2}+\frac{h_{a}+h_{e}}{2})\\ & +\alpha_{1}f_{a}h_{a}^{'}(b_{f}^{'}+b_{e})(h_{w0}-\frac{x}{2}+\frac{h_{a}}{2}+h_{e})+f_{y}^{'}A_{s}^{'}(h_{w0}-a_{s})+f_{ya}^{'}A_{sa}^{'}(h_{w0}-a_{s}+a_{s}^{'}+\frac{h_{a}^{'}}{2}+h_{e}^{'})\\ & +f_{ya}A_{sa}(a_{s}+\frac{h_{a}}{2}+h_{e})-\frac{1}{2}f_{yw}b_{w}\rho {\left(h_{w0}-1.5x\right)}^{2}\\ & -\frac{1}{2}f_{ya}b_{a}\rho (h_{w0}-1.5x+a_{s}+h_{e})(h_{w0}-1.5x-a_{s}-h_{e}) \end{array}$$

Among them, $e=e_{0}+h_{w0}-\frac{h_{w}}{2},e_{0}=M/N,x\geq 2a_{s}^{'}$ and the horizontal load-bearing capacity of the wall can be calculated as follows:

(4) $$F=\frac{M}{H}=\frac{Ne_{0}}{H}$$

The wall of SW1.5-1 and SW1.5-3 did not have the FAC outer protective layer, and symmetrical reinforcement was used for specimens ($f_{y}^{'}=f_{y},A_{s}^{'}=A_{s},a_{s}^{'}=a_{s}$). Thus, the formula can be simplified to:

(5) $$N=\alpha_{1}f_{c}b_{w}x+\alpha_{1}f_{c}(b_{f}^{'}-b_{w})h_{f}^{'}-f_{yw}b_{w}\rho (h_{w0}-1.5x)$$

(6) $$Ne=\alpha_{1}f_{c}b_{w}x(h_{w0}-\frac{x}{2})+\alpha_{1}f_{c}h_{f}^{'}(b_{f}^{'}-b_{w})(h_{w0}-a_{s})+f_{y}^{'}A_{s}^{'}(h_{w0}-a_{s})-\frac{1}{2}f_{yw}b_{w}\rho {\left(h_{w0}-1.5x\right)}^{2}$$

The horizontal load-bearing capacity *F* of SW1.5-2 shear wall can be calculated using Equations (2)–(4). The horizontal load-bearing capacity *F* of SW1.5-1 and SW1.5-3 is calculated by the Equations (4)–(6). Table 8 shows the *MV* and calculated value of horizontal load-bearing capacity *F* of the shear wall. The results show that the error between *MV* and calculated value of SW1.5-1 and SW1.5-3 is <5%. However, the error in SW1.5-2 is relatively large, mainly because of the relatively low load-bearing capacity for the base cracking during the test. Overall, the calculation results are in good agreement with the experimental results.

#### 4.1.2. Analysis of Load-Bearing Capacity of the Cross Section

The shear features of inclined section were mainly reflected from the load-bearing capacity of the wall with a shear-span ratio of 0.8. Therefore, it was necessary to establish the calculation formula for the load-bearing capacity of low-rise walls. The basic assumption of the oblique section is as follows:

The shear force was undertaken by recycled concrete and the web distribution reinforcement of the wall [28],

(7) $$V=V_{C}+V_{S}$$

For the shear force $V_{C}$, which was undertaken by recycled concrete, the action of shear zone was considered,

(8) $$V_{c}=0.5f_{t}b_{w}h_{w0}+\frac{0.28}{1+0.6(H/h_{w})}N\frac{A_{w}}{A}$$

The shear force in the web of the low-rise wall was undertaken by the horizontal and vertical distribution reinforcement.

(9) $$V_{s}=\frac{{\left(H/h_{w}\right)}^{4/3}}{1+{\left(H/h_{w}\right)}^{2/3}}\eta \rho_{sh}\omega_{1}f_{yh}b_{w}h_{w}+\frac{1}{1+{\left(H/h_{w}\right)}^{2/3}}\eta \rho_{sv}\omega_{2}f_{yv}b_{w}h_{w}$$

(10) $$\omega_{1}=\frac{1}{\sqrt{1+3{\left(H/h_{w}\right)}^{2/3}}}$$

(11) $$\omega_{2}=\frac{1}{\sqrt{3+{\left(H/h_{w}\right)}^{2/3}}}$$

In the formula, $f_{t}$ is the axial compressive strength of the recycled concrete; $b_{w}$ is the thickness of the web of the recycled concrete wall; $h_{w}$ is the total width of the recycled concrete wall along the shear direction; $h_{w0}$ is the clear width of the recycled concrete wall along the shear direction; N is the vertical pressure of the wall section; A is the total area of the recycled concrete wall; $A_{w}$ is the net area of the recycled concrete wall along the shear direction; H is the calculation value of the height of the recycled concrete wall; $\rho_{sh}$ and $\rho_{sv}$ are the reinforcement ratio of the horizontal and vertical distribution bar of the wall, respectively; $f_{yh}$ and $f_{yv}$ are the yield strength of the horizontal and vertical distribution reinforcement of wall, respectively; $\omega_{1}$ and $\omega_{2}$ are the stress coefficient of the horizontal and vertical distribution reinforcement, respectively; η is the shear friction coefficient among the aggregates on the diagonal section of the recycled concrete and is taken as 0.85 in this study.

The horizontal load-bearing capacity *F* of three shear walls was calculated using Equation (7). Table 9 shows the comparison between the *MV* and calculated value of the horizontal load-bearing capacity of the shear wall. Table 9 shows that the *MV* is in good agreement with the calculated value. The error between the *MV* and calculated value of all the three walls was <5%, and the accuracy of the calculation formula for the normal section was verified at the same time.

### 4.2. Finite Element Analysis

Further damage and evolution of recycled concrete was analyzed using a microscope. Under a low cyclic loading, the skeleton curve of the shear wall was similar to the load displacement curve under monotonic loading. Therefore, the performance of low cycle loading was simulated by using the monotonic loading. In this study, the damage and plastic strain of SW1.5-1 wall concrete was analyzed using the finite element software ABAQUS (ABAQUS Inc., Pawtucket, RI, USA), and the crack distribution, damage process, and mechanical characteristics of the specimen were discussed. The calculation results were compared with the experimental results.

#### 4.2.1. Finite Element Parameter

The concrete plastic damage model was used to simulate the recycled concrete and FAC. The constitutive relationship between stress and strain under the uniaxial pressure of recycled concrete proposed by Xiao [29] was used as shown in Formula (12):

(12) $$y=\{\begin{array}{c} ax+(3-2a)x^{2}+(a-2)x^{3},0\leq x<1\\ \frac{x}{b{\left(x-1\right)}^{2}+x},x\geq 1 \end{array}$$

where $x=\varepsilon /{\varepsilon_{0}}^{\prime },y=\sigma /{f_{c}}^{\prime }$; a is the coefficient at the ascending stage of the curve and $a=2.2(0.748r^{2}-1.231r+0.957)$, taken as *a* = 1.0428; b is the coefficient at the descending stage of the curve and b=0.8(7.6483r+1.142), taken as *b* = 7.03224. The peak compressive strain of the recycled concrete prism is $\varepsilon_{0}^{r}$, 1.2 times the peak compressive strain of ordinary concrete [29], and $f_{c}^{r}$ is the compressive strength of recycled concrete prism and taken as, $f_{c}^{r}=0.67f_{cu}$.

The model hypothesis of the tensile stress-strain law is concrete without damage before reaching the peak stress. The descending curve was calculated using Equation (13).

(13) $$y=\frac{x}{\alpha_{t}{\left(x-1\right)}^{2}+x},x\geq 1$$

where $x=\varepsilon /\varepsilon_{t0}^{r}$, $y=\sigma /f_{t}^{r}$; $\varepsilon_{t0}^{r}$ is the peak tensile strain of the recycled concrete prism. $\varepsilon_{t0}^{r}=f_{t}^{r}/E_{c}$; $f_{t}^{r}$ is the representative value of the uniaxial tension strength of concrete. $f_{t}^{r}=(-0.06r+0.24){\left(f_{cu}^{r}\right)}^{2/3}$; $\alpha_{t}^{r}$ is the coefficient at the descending stage of the uniaxial tension stress-strain curve of concrete.

The “plasticity” model from the ABAQUS software was used to simulate the reinforcement law. The Mises yield and the kinematic hardening criteria were used in the numerical simulation. The stress-strain relationship of the steel bar was simplified as the oblique line. In this study, ${E^{\prime }}_{s}=0.01E_{s}$ [27], and the Poisson’s ratio was 0.3.

#### 4.2.2. Finite Element Model

**Figure 19:**
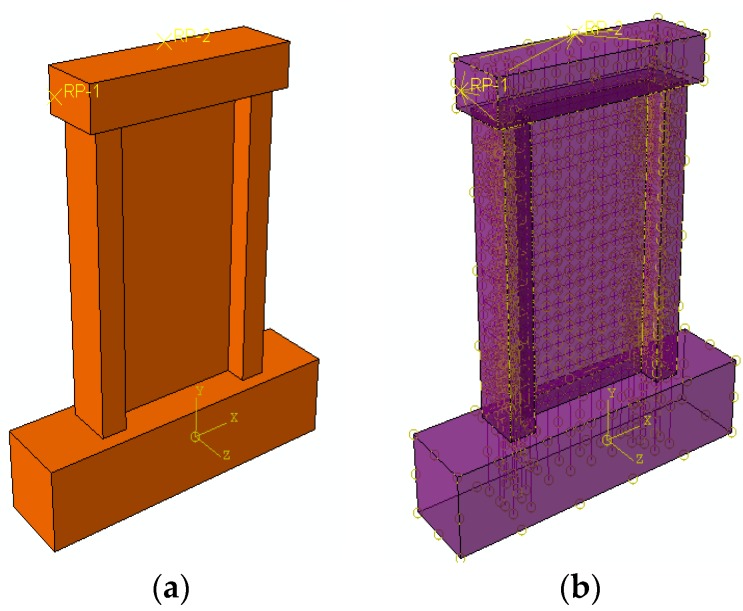
Establishment of model. (**a**) Assembled model; (**b**) Interaction of model.

The 3D solid element C3D8R was used to simulate the recycled concrete, and the 3D truss element T3D2 was used for the rebar. The separating method was used to create the components from different steel, and the material and section properties were defined later. The reinforced skeleton was embedded into the recycled concrete wall, and then the interaction of the model was defined as shown in Figure 19.

**Figure 20:**
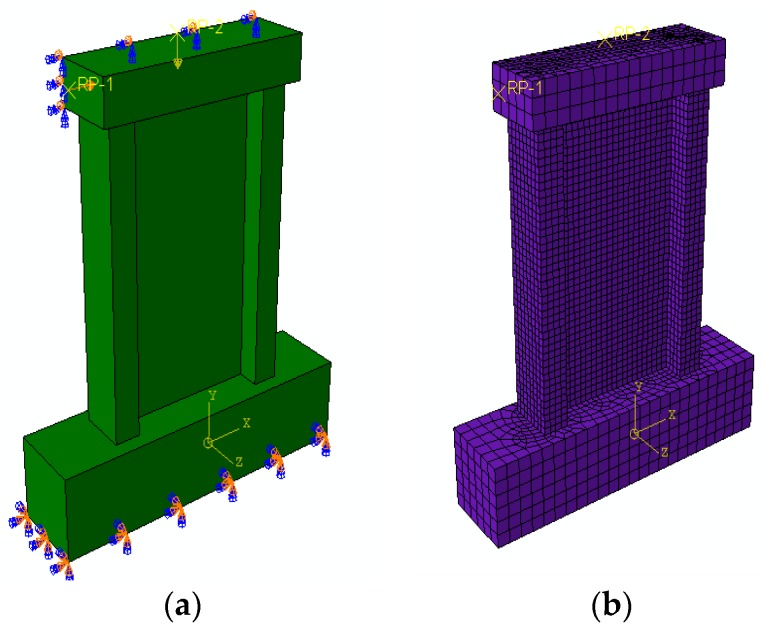
Boundary condition and mesh of the model. (**a**) Boundary condition of model; (**b**) Element Mesh of model.

The base of the wall was fixed on the ground, and the load surface of the loading beam was restrained. First, a vertical load was applied to the reference points on the load beam and remained unchanged during the process. Second, the horizontal displacement was controlled to simulate the horizontal loading process by defining different boundary conditions. The wall model was divided by the structure mesh method. The boundary conditions and the grid division of the model are shown in Figure 20.

#### 4.2.3. Calculation Results Analysis-Damage Evolution

**Figure 21:**
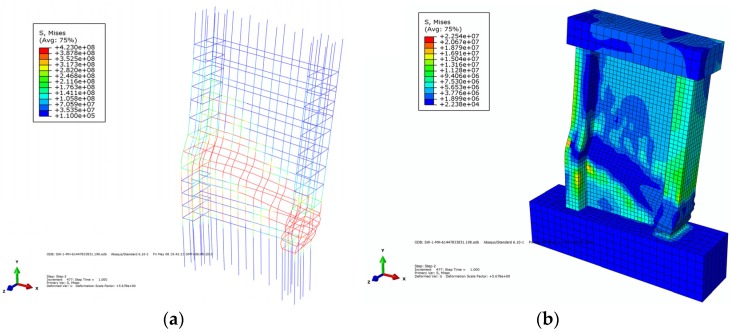
Steel stress of the model. (**a**) Stress cloud of the steel; (**b**) Stress cloud of the concrete.

**Figure 22:**
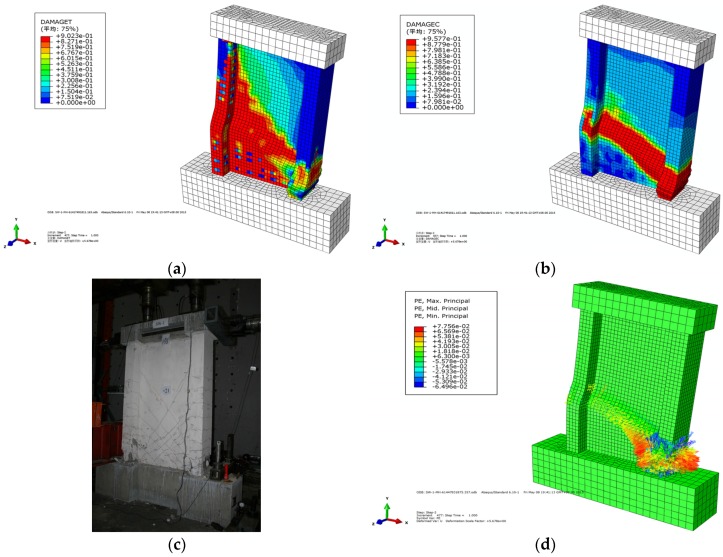
Concrete crack distribution of the model. (**a**) Pulling damage; (**b**) Compression damage; (**c**) Actual damage; (**d**) Vector map of maximum plastic strain.

When a tensile plastic strain appeared on the recycled concrete, it signified that the concrete element was cracked, and the width of the crack was reflected by the length of the vector arrow of the maximum plastic strain. The crack distribution was reflected by the vector map of the maximum plastic strain and the tensile damage of the concrete. Figure 21 shows the reinforced stress cloud and concrete stress cloud when the wall of SW1.5-1 reached the ultimate displacement. Figure 22a–c shows the pulling damage, compression damage, and actual damage of SW1.5-1. The vector map of the maximum plastic strain of SW1.5-1 is shown in Figure 22d.

The following conclusions can be drawn from Figure 21 and Figure 22:

(1) The cracks on the wall of SW1.5-1 mainly appeared on the middle and lower part of the flange and web, and the horizontal and diagonal cracks appeared on the flange and web of the wall, respectively.
(2) When the wall of SW1.5-1 reached the ultimate displacement, compressional damage occurred at the bottom of the web and flange, whereas tensional damage occurred at the lower part of the flange, exhibiting the damage characteristics of bending failure. The simulation phenomena are consistent with the experiment.
(3) The maximum stress of the steel bar appeared at the bottom of the longitudinal reinforcement on the compression side when the wall of SW1.5-1 reached the ultimate displacement of the test. At the same time, the longitudinal steel of the pull side yielded.

#### 4.2.4. Calculation Result Analysis-Skeleton Curve

**Figure 23:**
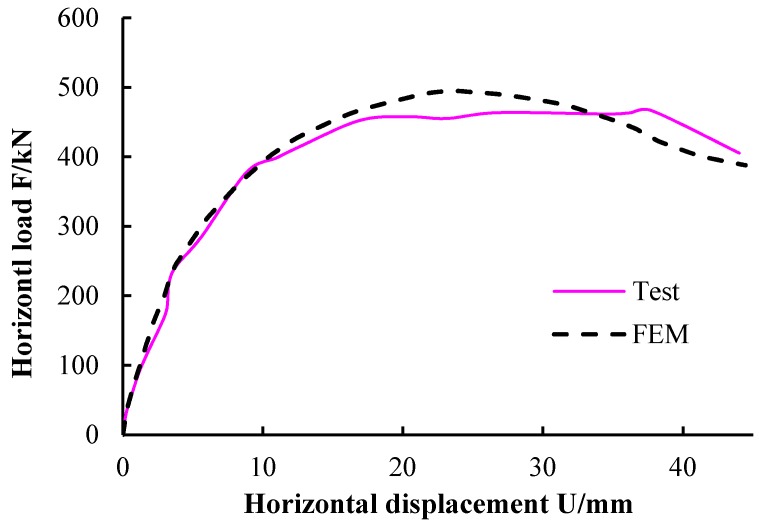
Comparison between measured and calculated curves of “*F*–*U*”.

The model was calculated using the Newton–Raphson method. The calculated curves “F horizontal load–U horizontal displacement” was compared to the measured skeleton curve, as shown in Figure 23. The skeleton curve of the numerical simulation is in good agreement with the measured skeleton curve, and the calculated ultimate load-bearing capacity is slightly lower than that of the finite element model.

## 5. Discussion and Suggestions on Experimental Design

Because of the limitation of experimental conditions, the vertical and horizontal loadings in the entire test process were controlled artificially; thus, the data of the hysteresis curves fluctuated slightly. Therefore, it is recommended to select a controlling system to control the loading process if the condition allows, making the curve smoother. During the experiment, the early cracking of the foundation of the individual components significantly affected the test results. This is mainly because once the foundation cracked, the foundation became relatively slippery. Therefore, the horizontal load cannot be reflected in the real component, and the final result of the test data is less. Therefore, the foundation and loaded beam should be designed to be stronger than the component; thus, the test target can be damaged first. On the whole, the results of this experiment satisfy the requirements of accuracy and error.

The composite shear wall with the EPS module is suitable for cold regions with high requirements for heat preservation and energy saving, and the structure also has a good seismic performance. In the case of a low shear-span ratio, the seismic performance of the composite shear wall with the EPS module is better. Moreover, a high load-bearing capacity makes it suitable for the seismic design of low-rise buildings.

Considering fire prevention, the composite wall with a FAC protective layer is more reliable. The EPS module of the composite shear wall should be uniform and symmetrically arranged. For the design of a composite shear wall, a gap of 10–15 mm should be used if the EPS module cannot be split at the junction (such as the junction between the basis and EPS module), and then the gap should be sealed using a polyurethane foam.

For the composite shear wall with a FAC protective layer, a deeper dovetail groove for the EPS module would be better. The FAC protective layer and shear wall body should be well connected using a connecting bar, and the layer should be arranged as a wire mesh. The reinforcement ratio should be consistent with the ratio of the shear wall.

## 6. Conclusions

In this paper, the seismic performance of a recycled concrete composite shear wall with EPS modules was studied. The main conclusions are as follows:

(1) Compared to an ordinary recycled concrete shear wall, the shear strength of the composite shear wall with EPS modules significantly improved, the speed of stiffness degradation slowed down, the hysteresis loops were relatively full, and the energy dissipation capacity improved.
(2) The seismic performance of the shear wall with an EPS sandwich layer was better than that with an EPS module-FAC outer protective layer. The horizontal load-bearing capacity and stiffness of the shear wall with the EPS sandwich layer clearly improved, and the ductility and energy dissipation of the wall were also enhanced. Although the FAC outer protective layer contributed less to the seismic performance of the wall, it could slow down the speed of cracking and stiffness degradation of the wall.
(3) Based on the experimental study, considering the structural characteristics of an EPS module, calculation formulas are proposed for the load-bearing capacity of the normal/oblique section of the composite shear wall with an EPS module. The calculation results are in good agreement with the experimental results.
(4) Using the ABAQUS finite element software, a finite element model was developed for the shear wall without an EPS module. The damage evolution of the wall was analyzed. The skeleton curve of the finite element model is in good agreement with the experimental results.
(5) In the case of a low shear-span ratio, the seismic performance of the recycled concrete composite shear wall was better, and the load-bearing capacity was reliable. The symmetrical layout of EPS modules in the composite shear wall should be given priority, and a composite wall with a FAC protective layer would be better for fire resistance. The FAC protective layer should be arranged using a steel wire mesh, and it should be reliably connected to the wall.

## Figures and Tables

**Table 1:** Experimental results of concrete mechanical properties. RAC = Recycled aggregate concrete, FAC = Fine aggregate concrete.

Type of Concrete	Concrete Strength	Cube Compressive Strength (MPa)	Elastic Modulus (MPa)
RAC	RC40	42.6	32,958
FAC	C40	41.8	31,553

**Table 2:** Physical properties of expanded polystyrene (EPS) module.

Physical Properties	Value
Apparent density (kg/m^3^)	18–22
Thermal conductivity (W/(m·K))	≤0.039
Compressive strength (MPa)	≥0.10
Tensile strength of the plate direction (MPa)	≥0.10
Dimensional stability (%)	≤0.50
Water absorption (%)	≤4

**Table 3:** Mechanical properties of steel bars.

Grade Level	Diameter (mm)	*f_y_* (MPa)	*f_u_* (MPa)	δ (%)	*E_s_* (MPa)
HPB235	4	253.35	359.36	34.1	1.95 × 10^5^
HPB300	6	386.33	578.33	21.6	2.05 × 10^5^
HRB400	8	462.33	699.67	17.6	2.01 × 10^5^
HRB400	12	423.00	598.33	23.1	2.02 × 10^5^

**Table 4:** Dimension and reinforcement details of specimens.

Specimen	SW1.5-1	SW1.5-2	SW1.5-3	SW0.8-1	SW0.8-2	SW0.8-3
Height (mm)	2100	2100	2100	1050	1050	1050
Thickness (mm)	200	320	320	200	320	320
Shear-span ratio	1.5	1.5	1.5	0.8	0.8	0.8
Steel ratio	0.25%	0.25%	0.25%	0.25%	0.25%	0.25%
Vertical web rebar	D8-200	D8-200	D8-160	D8-200	D8-200	D8-160
Horizontal web rebar	D8-200	D8-200	D8-160	D8-200	D8-200	D8-160
Concealed column rebar	4D12	4D12	4D12	4D12	4D12	4D12
Steel net	-	D4-10	-	-	D4-10	-
Stirrup	D6-150	D6-150	D6-150	D6-150	D6-150	D6-150
Connecting bar	D6	D6	D6	D6	D6	D6

**Table 5:** Experimental results of cracking load, yield load, and ultimate load.

Specimen	*F_c_* (*kN*)		*F_y_* (*kN*)		*F_u_* (*kN*)		μ*_cu_*	μ*_yu_*
	*MV*	*RV*	*MV*	*RV*	*MV*	*RV*	*F_c_/F_u_*	*F_y_/F_u_*
SW1.5-1	60.80	1.000	422.43	1.000	481.5	1.000	0.126	0.877
SW1.5-2	59.05	0.971	388.28	0.919	450.5	0.936	0.131	0.862
SW1.5-3	61.68	1.014	428.29	1.014	516.3	1.072	0.119	0.830
SW0.8-1	180.47	1.000	688.26	1.000	830.43	1.000	0.217	0.829
SW0.8-2	213.12	1.181	782.41	1.137	901.36	1.085	0.236	0.868
SW0.8-3	204.96	1.136	739.84	1.075	892.62	1.075	0.230	0.829

**Table 6:** Stiffness and degeneration coefficient of specimens.

Specimen	*K*_0_ (kN∙mm^−1^)		*K_y_* (kN∙mm^−1^)		β*_y_*_0_ = *K_y_*/*K*_0_	
	*MV*	*RV*	*MV*	*RV*	*MV*	*RV*
SW1.5-1	129.8	1.000	35.8	1.000	0.276	1.000
SW1.5-2	215.0	1.656	76.5	2.138	0.356	1.289
SW1.5-3	251.2	1.935	72.0	2.010	0.287	1.038
SW0.8-1	463.65	3.572	67.5	1.885	0.146	0.529
SW0.8-2	524.33	4.040	103.1	2.880	0.197	0.714
SW0.8-3	563.21	4.339	123.5	3.450	0.219	0.793

**Table 7:** Experimental results of energy dissipation.

Specimen	*E*_0.02_ (kN·mm)	*RV*	Specimen	*E*_0.02_ (kN·mm)	*RV*
SW1.5-1	49,529	1.000	SW0.8-1	195,206	1.000
SW1.5-2	52,154	1.053	SW0.8-2	278,420	1.426
SW1.5-3	54,285	1.096	SW0.8-3	319,057	1.634

**Table 8:** Mechanical and experimental results of ultimate load.

Specimen	*MV* (kN)	Calculated Value (kN)	Relative Error
SW1.5-1	481.5	466.3	−3.16%
SW1.5-2	450.5	514.5	14.21%
SW1.5-3	516.3	503.6	−2.46%

**Table 9:** Calculated and experimental results of ultimate load.

Specimen	*MV* (kN)	Calculated Value (kN)	Relative Error
SW0.8-1	830.43	805.28	−3.03%
SW0.8-2	901.36	860.36	−4.55%
SW0.8-3	892.62	934.61	4.70%

## Abbreviations

The following abbreviations are used in this manuscript:

EPS: Expandable polystyrene
RCA: Recycled concrete aggregate
FAC: Fine aggregate concrete
*MV*: Measured value
*RV*: Relative value
